# Association Analysis of SNPs in *GRHL2* and *RORA* Genes with Lambing Number in Small-Tailed Han Sheep

**DOI:** 10.3390/ani15101432

**Published:** 2025-05-15

**Authors:** Xiufen Pu, Kai Liu, Xiangyu Wang, Ran Di, Xiaoyun He, Yufang Liu, Mingxing Chu

**Affiliations:** State Key Laboratory of Animal Biotech Breeding, Institute of Animal Science, Chinese Academy of Agricultural Sciences, Beijing 100193, China; pxf182324@163.com (X.P.); lk2000oo@163.com (K.L.); wangxiangyu@caas.cn (X.W.); diran@caas.cn (R.D.); hexiaoyun@caas.cn (X.H.)

**Keywords:** sheep, lambing number, *GRHL2*, *RORA*, SNPs

## Abstract

Previous studies on the *GRHL2* and *RORA* genes were mainly focused on hormone regulation and early embryonic development. However, studies investigating the relationship between these genes and reproductive traits, such as lambing number, have been relatively limited. This study explored the associations between four single-nucleotide polymorphisms (SNPs) of the *GRHL2* gene (rs402967440, rs411785026, rs424408342, and rs411712725), one SNP (rs425540123) of the *RORA* gene, and the lambing number at the first, second, and third parities, as well as the average lambing number in Small-tailed Han sheep. Sanger sequencing revealed that all five SNPs were distributed in the Small-tailed Han sheep population. Subsequently, Agena Bioscience MassARRAY technology was used to genotype these five SNPs in the 384 Small-tailed Han sheep and analyze their population genetic characteristics, as well as their associations with the lambing number. The population genetic analysis showed that the rs402967440, rs424408342, and rs411712725 loci in *GRHL2* and the rs425540123 locus in *RORA* exhibited low genetic polymorphism in the Small-tailed Han sheep population (*PIC* < 0.25), while the *GRHL2* mutation rs411785026 showed moderate polymorphism (0.25 < *PIC* < 0.5). The *GRHL2* mutations rs402967440 and rs424408342 were not in Hardy–Weinberg equilibrium (*p* < 0.05), whereas the *GRHL2* mutations rs411785026 and rs411712725 and the *RORA* mutation rs425540123 were in Hardy–Weinberg equilibrium (*p* > 0.05). An association analysis showed that only the rs402967440 locus of the *GRHL2* gene was significantly associated with the lambing number trait of the Small-tailed Han sheep (*p* < 0.05). To further elucidate the potential role of GRHL2 in reproduction, the STRING protein interaction database was further used to predict the interaction network between the *GRHL2* protein and the reproduction-related proteins. It was found that *GRHL2* interacted with the candidate genes *OVOL2* and *VWC2L*, which are involved in the regulation of the reproductive process. These results indicate that *GRHL2* can be used as a novel genetic marker for the breeding of polytocous sheep. It also has the potential to improve the economic benefits of the sheep industry by regulating the lambing number.

## Simple Summary

*GRHL2* and *RORA* have been previously screened as candidate genes related to the reproductive traits of sheep. Therefore, it is necessary to verify the relationship between these two genes and the lambing number trait in sheep. The results of this study showed that the rs402967440 locus of *GRHL2* was significantly associated with the lambing number in sheep, providing a baseline reference for the molecular breeding of polytocous sheep.

## 1. Introduction

As one of the essential reproductive traits in sheep, the lambing number directly affects the economic benefits of the sheep breeding industry [1]. Traditional breeding methods have limitations such as long cycles and low efficiency in improving the multiple lambing trait in sheep [2]. Molecular marker screening, as a modern biotechnological means, can effectively overcome the shortcomings of traditional breeding and significantly enhance the efficiency of genetic improvement for the multiple lambing trait in sheep [3,4]. Marker-assisted selection technology can directly reflect the differences at the DNA level between species. Moreover, it is not affected by environmental factors, developmental stage, tissue type, or other variables, making it stable and reliable. Using molecular markers related to quantitative traits, genetic structure can be accurately and quickly analyzed at the molecular level to select patterns and improve breeding efficiency [5]. iPLEX technology has been widely used in the identification of molecular genetic markers, breeding of new strains, genome-wide association analysis, and improving production performance, showing its important application value and prospects in modern animal husbandry [6]. Genome-wide association analysis (GWAS) has been used to identify candidate genes associated with reproductive traits in sheep. These studies have found that certain genes, such as bone morphogenetic protein 15 (BMP15), growth differentiation factor 9 (GDF9), and bone morphogenetic protein receptor 1B (BMPR1B), have a significant effect on sheep fertility [7,8,9]. The use of these molecular markers for assisted selection can improve the reproductive efficiency of sheep breeds and achieve higher productivity and economic benefits [10]. Therefore, it is necessary to further understand the molecular mechanism of the effect of more candidate genes on the lambing number of sheep and the regulation network of multiple genes on the lambing number, as well as promote the economic benefits of the sheep industry.

Grainyhead-like transcription factor 2 (*GRHL2*) is a member of the granular head (GRHL) protein family [11], which mainly includes *GRHL1*, *GRHL2(BOM)*, and *GRHL3* (*SOM*) and has highly conserved functions in drosophila and vertebrates. It is an important transcription factor for organ development [12], epithelial differentiation repair [13], and barriers [14]. Each member has a unique function. *GRHL2* regulates epithelial molecular plasticity [13], participates in the regulation of midbrain–posterior brain boundary pattern and morphology [15], and participates in tissue fusion [16]. Studies have shown that *GRHL2* regulates the expression of a variety of genes and participates in a variety of physiological processes, such as cell proliferation [17], migration, and invasion [18]. The expression of *GRHL2* varies across different tissues. *GRHL2* can be a tumor suppressor or carcinogenic factor [19]. *GRHL2* can play a role in regulating cell proliferation and apoptosis through PI3K/Akt pathway-related proteins [20], and it also affects cell invasion and migration by inhibiting the induction of TGF-β [21]. Studies have shown that *GRHL2* is a key regulator of embryonic development in mammals. In mouse embryo research, *GRHL2* was found to coordinate a gene network controlling trophoblast branching morphogenesis and thus promoting embryo development [22]. Estrogen can enhance *GRHL2* binding to phosphorylated ER [23], and *GRHL2* can enhance the phosphorylation of estrogen receptor DNA binding regulation and its mediated transcriptional activation and repression functions [24]. A lack of *GRHL2* may cause embryonic lethality [16], abnormal tissue fusion [25], and the closure of the ectodermal neural tube [26]. Though *GRHL2* is little studied in sheep, it plays a crucial role in cell proliferation, embryo development, and estrogen regulation, affecting reproduction and possibly impacting sheep reproductive traits.

Retinoic acid-related orphan receptor alpha (*RORA*) belongs to the steroidogenic nuclear hormone receptor superfamily, binds to ROR response elements (ROREs) as a transcription factor in the regulatory region of the target gene as a monomer or homodimer, and participates in a variety of physiological processes, such as the regulation of circadian rhythms [27], inflammation [28], metabolism [29], and cell development [30]. *RORA* was identified as a potential marker of oocyte maturation in the study of transcription and regulation of macromolecular metabolic processes in cultured porcine oocytes in vitro [31]. There is a significant reduction in the risk of placental abruption at *RORA*, with some favorable effects on reproduction [32]. At the same time, it was found that *RORA* was expressed in the ovarian epithelium of goats [33], and it is the transcriptional target of estrogen receptors, regulates the synthesis of estrogen [34], and regulates the aging of human cumulus cells [35]. *RORA* was found to be associated with lambing number in sheep in a study of Australian white sheep [36]. These results showed that *RORA* was closely related to the regulation of hormone synthesis and function of the ovary and had an effect on lambing number in sheep. *GRHL2* and *RORA* play crucial roles in reproduction and related hormone regulation, but their potential impacts on sheep reproduction are not yet understood. Therefore, this study focuses on exploring the effects of SNP mutations in the *GRHL2* and *RORA* genes on lambing number and gene function in sheep. We analyzed the association between these SNP sites and lambing number and used bioinformatics to predict protein interaction networks, revealing the potential functional impacts of the SNPs. This research may provide valuable molecular markers for sheep breeding.

## 2. Materials and Methods

### 2.1. Ethical Approval

This study was conducted in accordance with the Guidelines for Experimental Animals Established by the Ministry of Science and Technology (Beijing, China). All experimental protocols were approved by the Science Research Department (in charge of animal welfare issues) of the Institute of Animal Science, Chinese Academy of Agricultural Sciences (CAAS, Beijing, China) (No. IAS 2020-82).

### 2.2. Animal Preparation and Sample Collection

This experiment involved 384 ewes from Small-tailed Han sheep; all ewes were around three years old. Small-tailed Han sheep are known for their polytocous trait, which contributes to higher lambing numbers. Lambing number refers to the number of lambs produced in a litter during sheep reproduction. It serves as a key indicator of the reproductive performance of sheep, reflecting the reproductive ability and productivity of the ewe. The lambing number of the Small-tailed Han sheep was specifically recorded for three parties between 2017 and 2020 (Supplementary Table S1). All sheep were housed in the same facilities, ensuring a nutritious diet and reducing any associated experimental manipulations that might cause stress. None of the ewes carried the *FecB* gene. Blood samples of all sheep samples were collected through the jugular vein and stored at −20 °C using EDTA anticoagulant.

### 2.3. Blood DNA Extraction

DNA was extracted from blood samples using the instructions in the DNA extraction kit (Tiangen Biochemical Technology Co., Ltd., DP304-03, Beijing, China). A NanoDrop 2000 was used to determine the concentration of the extracted DNA samples, and 1.5% agarose gel electrophoresis was performed to evaluate the quality of the extracted DNA samples.

### 2.4. PCR Amplification and Sanger Sequencing

Based on the results of the Ensembl database (Oar_v3.1) annotations and previous resequencing data, different SNPs in the *GRHL2* and *RORA* genes were amplified by PCR, and three DNA samples per gene SNP were taken for Sanger sequencing. We designed primers with Primer Premier 5 to amplify the products. The primer sequences are listed in Table 1. The primers were synthesized by Shanghai Bioengineering Biotechnology Service Co., Ltd. (Shanghai, China). The PCR products were analyzed via agarose gel electrophoresis and Sanger sequencing, with the latter performed by Shanghai Bioengineering Biotechnology Service Co., Ltd.

The PCR reaction mixture (25 μL) comprised 12.5 μL of 2×Master Mix (KT201-02, Tiangen Biotech Co., Ltd.), 1 μL each of upstream and downstream primers (10 μM), 1.56 μL of DNA template, and 8.94 μL of ddH_2_O. The amplification program included an initial denaturation at 94 °C for 3 min, followed by 30 cycles of denaturation at 94 °C for 30 s, annealing at 60 °C for 30 s, and extension at 72 °C for 1 min, with a final extension at 72 °C for 5 min.

### 2.5. Genotyping

Based on the sequences of sheep *GRHL2* and *RORA* genes in GenBank ARS-UI_Ramb_v2.0 (accession Nos: NC_056062.1 and NC_056060.1), single-base extension primers for five loci (g.75350274G > A, g.75351375T > C, g.75351555G > A, and g.46741101A > T of *GRHL2* and g.46741101A > T in *RORA*) were designed with MassARRAY Assay Design v.3.1. The sequences of these primers are shown in Table 2, which were synthesized by Beijing Compass Biotechnology Co., Ltd. (Beijing, China).To investigate the distribution of *GRHL2* and *RORA* in the Small-tailed Han sheep population, we used the iPLEX technology (Agena Bioscience, San Diego, CA, USA) for genotyping and related sequencing results; we referred to previous studies for the detailed process of genotyping [37,38].

### 2.6. Data Analysis

Genotyping data were used to calculate allele and genotype frequencies, polymorphism information content (*PIC*), heterozygosity (*HE*), the effective number of alleles (*NE*), and *p*-values (*chi-square test*). With a chi-square test value of *p* > 0.05, it was considered that the ewe population was in Hardy–Weinberg equilibrium. To determine the association between genotype and lambing number, an adjusted linear model was applied:

$$y_{ijn}=\mu +P_{i}+G_{j}+I_{PG}+e_{ijn}$$

where $y_{ijn}$ is the phenotypic value (lambing number); *μ* denotes the group average; $P_{i}$ represents the fixed effect of the ith parity (i = 1,2 or 3); $G_{j}$ represents the effect of the jth genotype (j = 1, 2 or 3); $I_{PG}$ is the interaction effect of parity and genotype; and $e_{ijn}$ represents random error. For the data analysis, Microsoft Excel 2016 was employed to calculate allele and genotype frequencies, *PIC*, *HE*, and *NE* and conduct chi-square tests for Hardy–Weinberg equilibrium (*p* > 0.05 indicates equilibrium). IBM SPSS Statistics 27 (SPSS Inc., Chicago, IL, USA) was then utilized to perform an ANOVA and evaluate the correlation between genotypes and lambing numbers.

### 2.7. Using STRING Database to Predict Protein–Protein Interaction Network

To further understand the function of the candidate genes, we used STRING database v.11.0 (https://cn.string-db.org/, accessed on 11 April 2025) to analyze their network interactions with reproduction-related proteins.

## 3. Results

### 3.1. Identification of GRHL2 SNPs and RORA SNP Mutation Sites

**Figure 1:**
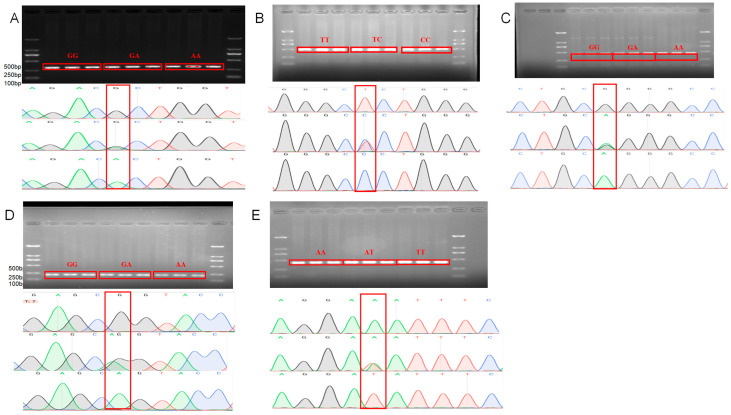
Gel electrophoresis and Sanger sequencing results of SNP loci in *GRHL2* and *RORA*: (**A**) *GRHL2* rs402967440; (**B**) *GRHL2* rs411785026; (**C**) *GRHL2* rs424408342; (**D**) *GRHL2* rs411712725; (**E**) *RORA* rs425540123. Red frames are marked with mutation points.

Based on SNP annotation, mutations in candidate SNP sites needed to be further identified. We used Sanger sequencing to confirm these mutations. The results showed that rs402967440, rs411785026, rs424408342, and rs411712725 in *GRHL2* and rs425540123 in *RORA* are all present in Small-tailed Han sheep, with the specific mutated bases at these positions illustrated in Figure 1. The results can be used to further study the distribution of SNP mutations in the Small-tailed Han sheep.

### 3.2. Genotyping and Population Genetic Analysis of Candidate SNPs in GRHL2 and RORA

**Figure 2:**
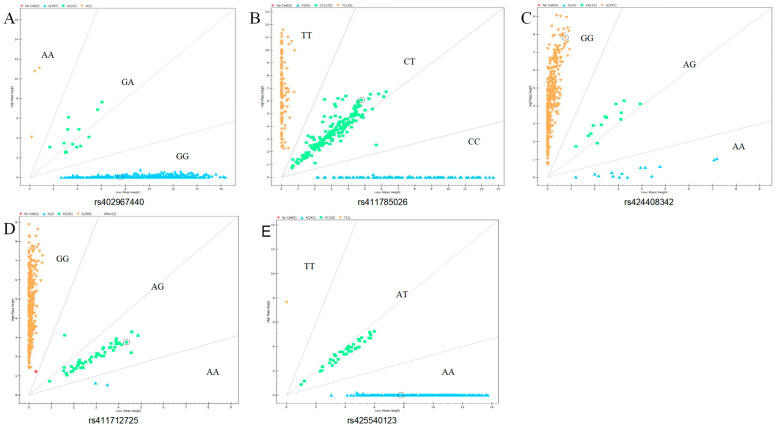
Genotyping results of candidate SNPs in *GRHL2* and *RORA* genes using MassARRAY SNP system: (**A**) rs402967440 of *GRHL2*; (**B**) rs411785026 of *GRHL2*; (**C**) rs424408342 of *GRHL2*; (**D**) rs411712725 of *GRHL2*; (**E**) rs425540123 of *RORA*.

To further analyze whether mutations in these SNPs affect the entire population of Small-tailed Han sheep, we performed genotyping and population genetic analysis. Genotyping of the candidate gene *GRHL2* (rs402967440, rs411785026, rs424408342, and rs411712725) showed that three genotypes exist in Small-tailed Han sheep: GG (n = 367.96%), GA (n = 14.4%), and AA (n = 3.1%) of rs402967440; TT (n = 119.31%), CT (n = 172.45%), and CC (n = 93.24%) of rs411785026; GG (n = 357.93%), GA (n = 13.3%), and AA (n = 14.4%) of rs424408342; and GG (n = 328.86%), GA (n = 52.14%), and AA (n = 2.1%) of rs411712725. Genotyping of *RORA* (rs425540123) showed the presence of three genotypes in the Small-tailed Han sheep: AA (n = 345.90%), AT (n = 38.10%), and TT (n = 1.0%) (shown in Figure 2). The population genetic analysis revealed that the *GRHL2* rs402967440 rs424408342, and rs411712725 and *RORA* rs425540123 mutation loci exhibited low genetic polymorphism (PIC < 0.25) in the Small-tailed Han sheep population, whereas the *GRHL2* rs411785026 mutation exhibited moderate polymorphism (0.25 < PIC < 0.5) (Table 3). The *GRHL2* mutations rs402967440 and rs424408342 were found not to be in Hardy–Weinberg equilibrium (*p* < 0.05), whereas the *GRHL2* mutations rs411785026 and rs411712725 and the *RORA* mutation rs425540123 were in Hardy–Weinberg equilibrium (*p* > 0.05) (Table 4).

### 3.3. Association Analysis of GRHL2 and RORA Candidate Loci with Lambing Number in Small-Tailed Han Sheep

The candidate genes were subjected to an association analysis with the lambing number of Small-tailed Han sheep (Table 4). The association analysis showed that the rs402967440 locus of *GRHL2* was significantly associated with the lambing number of the second parity (*p* = 0.046 < 0.05) and third parity (*p* < 0.001), as well as with the average lambing number (*p* = 0.002 < 0.01). No association was found in the first parity. The ewes with the GG genotype of the *GRHL2* gene had a significantly lower lambing number in the second and third parties than those with the GA and AA genotypes (*p* < 0.05). The analysis of the mean lambing numbers showed that the GA genotype had a higher mean lambing number than the other two genotypes. For the rs411712725 locus of *GRHL2*, a difference was observed between the AA and GG genotypes in the second parity. However, these data were deemed invalid and not discussed further due to a biologically implausible lambing number of zero for the AA genotype. Failure of ewes to conceive or lamb in the third lactation may be an effect of external environmental factors and ongoing management practices on reproductive performance in sheep. No significant association was found between the *RORA* rs425540123 mutation and lambing number (*p* > 0.05). Additionally, due to the limited sample size, the validity of these data could not be determined, and this gene will not be discussed further.

**Table 4:** Least squares mean and standard errors of lambing number in Small-tailed Han sheep with different genotypes.

Gene	SNPs	Genotypes	1st Parity Lambing Number	2nd Parity Lambing Number	3rd Parity Lambing Number	Average Lambing Number
*GRHL2*	rs402967440	GG (367)	1.98 ± 0.05	1.68 ± 0.07 ^a^	0.68 ± 0.07 ^a^	1.45 ± 0.05 ^a^
		GA (14)	2.36 ± 0.34	2.50 ± 0.39 ^b^	2.14 ± 0.53 ^b^	2.33 ± 0.34 ^b^
		AA (3)	2.00 ± 0.58	2.00 ± 0.58 ^ab^	1.33 ± 0.67 ^ab^	1.78 ± 0.22 ^ab^
*GRHL2*	rs411785026	TT (119)	2.09 ± 0.09	1.76 ± 0.13	0.70 ± 0.11	1.52 ± 0.08
		CT (172)	1.88 ± 0.08	1.71 ± 0.10	0.84 ± 0.11	1.49 ± 0.07
		CC (93)	2.09 ± 0.11	1.67 ± 0.15	0.61 ± 0.13	1.46 ± 0.10
*GRHL2*	rs424408342	GG (357)	1.99 ± 0.05	1.76 ± 0.07	0.77 ± 0.07	1.51 ± 0.05
		GA (13)	1.77 ± 0.28	1.15 ± 0.34	0.31 ± 0.24	1.08 ± 0.17
		AA (14)	2.36 ± 0.20	1.21 ± 0.35	0.36 ± 0.07	1.31 ± 0.17
*GRHL2*	rs411712725	GG (328)	1.98 ± 0.06 ^a^	1.76 ± 0.07 ^a^	0.78 ± 0.08	1.51 ± 0.05
		GA (52)	2.06 ± 0.15 ^a^	1.55 ± 0.19 ^a^	1.39 ± 0.18	1.39 ± 0.13
		AA (2)	2.67 ± 0.33 ^a^	0.00 ^b^	0.00	0.89 ± 0.11
*RORA*	rs425540123	TT (1)	3.00 ± 0.00	2.00 ± 0.00	0.00	1.67 ± 0.00
		AT (38)	1.89 ± 0.17	1.66 ± 0.23	0.89 ± 0.23	1.48 ± 0.16
		AA (345)	2.01 ± 0.05	1.72 ± 0.07	0.73 ± 0.07	1.49 ± 0.05

Note: Different letters represent a significant difference (*p* < 0.05). The numbers in parentheses represent the total number of ewes with that genotype.

### 3.4. GRHL2-Related Protein Interaction Network Prediction

**Figure 3:**
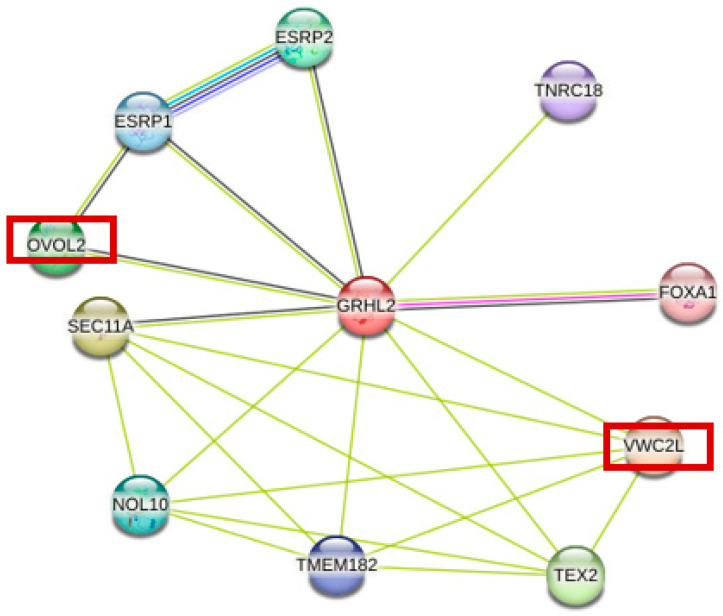
Protein network interacting with *GRHL2* proteins.

To further explore the biological functions of *GRHL2* in sheep reproductive traits, the STRING protein interaction network database was used to predict proteins interacting with *GRHL2*. Among the 10 proteins with the strongest interactions with *GRHL2* (Figure 3), key proteins closely related to the lambing numbers, such as VWC2L and OVOL2, were identified through interaction protein information (Table 5). These findings further indicate that *GRHL2* is an important factor affecting lambing number in sheep, and further validation of *GRHL2*’s role in sheep reproduction regulation will be needed in future studies.

## 4. Discussion

In this study, based on the preliminary sequencing data of several sheep breeds [37,39], five SNP loci were screened. The five mutation loci of the *GRHL2* and *RORA* genes were genotyped using iPLEX technology, and their correlation with the lambing number of Small-tailed Han sheep was further analyzed.

In this study, five single-nucleotide polymorphisms (SNPs) were successfully identified via Sanger sequencing. The five mutation loci of the candidate genes were genotyped using iPLEX technology. These findings firmly established the presence of these SNPs within the Small-tailed Han sheep population, providing a solid foundation for subsequent genetic analysis. The population genetic analysis yielded intriguing results regarding the genetic polymorphism of the identified SNPs. Specifically, the mutation loci, including *GRHL2* rs402967440, rs424408342, and rs411712725 and *RORA* rs425540123, exhibited low genetic polymorphism (PIC < 0.25) in the Small-tailed Han sheep population. This indicates limited genetic variation at these loci, which may have implications for the genetic diversity and adaptability of the breed. On the other hand, the *GRHL2* rs411785026 mutation showed moderate polymorphism (0.25 < PIC < 0.5), suggesting a relatively higher level of genetic variation at this locus. This moderate polymorphism could potentially contribute to the phenotypic diversity observed within the Small-tailed Han sheep population. Furthermore, the analysis of Hardy–Weinberg equilibrium revealed interesting patterns. The *GRHL2* mutations rs402967440 and rs424408342 deviated significantly from Hardy–Weinberg equilibrium (*p* < 0.05), indicating that certain evolutionary forces, such as selection, mutation, migration, or non-random mating, may be acting on these loci. In contrast, the *GRHL2* mutations rs411785026 and rs411712725 and the *RORA* mutation rs425540123 were found to be in Hardy–Weinberg equilibrium (*p* > 0.05), suggesting that these loci are relatively stable under the current population conditions and not subject to strong evolutionary pressures. In addition to the genetic analysis of the identified SNPs, a review of the existing literature on the functions of *GRHL2* provided valuable insights. In earlier studies, the *GRHL2* coordinated gene network was involved in the morphogenesis of embryonic trophoblast branching and promoted the development of the fetal mother exchange point [22]. The *GRHL2* gene not only acted as a key regulator of epithelial cells [14,20,40,41] but also was expressed in the ovarian epithelium [42] and was able to methylate and then affect the migration and invasion of cells [43] and regulate the cell growth, cell cycle, and metabolism [44], which was further hypothesized to be a possible regulator of the ovarian function.

In this study, we investigated the association of SNPs in the *GRHL2* and *RORA* genes with lambing numbers in Small-tailed Han sheep. Through the genotype analysis and lambing number association analysis, we found that the rs402967440 locus of the *GRHL2* gene was significantly correlated with lambing number, especially in the second and third litters. External factors may also have a potential impact on *GRHL2* functional expression. This suggests that *GRHL2* may be a potential genetic marker affecting lambing numbers in sheep breeding. More interestingly, *GRHL2* is involved in the regulation of estrogen receptor α, which binds to enhancers and regulates E2-mediated estrogen receptor phosphorylation [24,45]. In studying the effects of different photoperiods on the adrenal glands of Sunit sheep, it was found that *GRHL2*, as a key gene in photoperiod regulation, with increasing light duration may affect reproductive traits in sheep [46]. The role of *GRHL2* in reproduction-related signaling pathways provides a potential mechanism by which it affects litter size in Small-tailed Han sheep. *GRHL2* is involved in a variety of physiological processes and participates in signaling pathways, such as the PI3K/Akt pathway that regulates the cell cycle [20] and the PI3K/Akt pathway that can promote follicular activation through pharmacological or genetic regulation of reproduction [47]. The PI3K/Akt pathway is essential for oocyte maturation and embryo development in sheep [48,49,50]. *GRHL2* regulates the MAPK/TGF-β pathway in tumors, and activation of the MAPK/TGF-β pathway can drive oocyte maturation and development [51] TGF-β signaling plays a key role in follicular growth and development in sheep [52,53]. Follicular dynamics and embryonic development are key factors affecting the lambing number in Small-tailed Han sheep. This further suggests that *GRHL2* is involved in the reproductive process of sheep and may affect the lambing number of sheep by influencing ovarian hormones and embryonic development, which is consistent with the fact that *GRHL2* may affect the reproductive ability of sheep in this study. To understand the effect of *GRHL2* on the reproduction of sheep, this study analyzed the significant correlation between the mutation site and the lambing number of Small-tail Han sheep. The lambing number of mutant genotype GA was significantly higher than that of genotypes GG and AA. This further suggests that this gene may be involved in the regulation of reproduction to increase the lambing number of sheep through the signaling pathway mentioned above, which needs to be verified in future experiments.

To better understand the role of *GRHL2* in sheep reproduction, the *GRHL2* protein interaction network was analyzed. The results showed that the OVOL2 protein in the protein interaction network acts downstream of developmental signaling pathways such as Wg/Wnt and BMP/TGF-β to ensure early embryonic development by regulating the cell cycle [54] and that this signaling pathway affects the function of the ovary by regulating the secretion of inhibin B in ovarian granulosa cells [55], which is an important regulator of follicle formation [56]. VWC2L is a candidate gene for reproductive traits in Chinese Holstein cows and is associated with the conception rate in dairy cows [57], suggesting that both OVOL2 and VWC2L are involved in the regulation of the reproductive process. In summary, the *GRHL2* gene is associated with the regulation of lambing number in sheep. Therefore, the rs402967440 locus of *GRHL2* can be used as a potential molecular marker for lambing traits in Small-tailed Han sheep. In contrast to previous studies that mainly focused on the main effector genes, this study focused on the potential contribution of *GRHL2* and *RORA* microeffector polygenes in the regulation of reproductive traits and found that *GRHL2* may influence lambing number through its known functions in embryonic development and epithelial differentiation. The significant correlation between the rs402967440 locus of the GRHL2 gene and the lambing number suggests that this SNP could serve as a valuable molecular marker in sheep breeding programs. Incorporation of this marker into molecular-assisted selection strategies is expected to increase the lambing number and thus improve the economics of sheep production.

## 5. Conclusions

In conclusion, this study systematically analyzed the distribution of SNPs in the *GRHL2* and *RORA* genes of Small-tailed Han sheep and investigated their relationship with lambing numbers. It was found that the rs402967440 locus of the *GRHL2* gene was significantly correlated with the lambing number of the Small-tailed Han sheep, particularly in the second and third litters. Based on these findings, we suggest that the rs402967440 locus may be involved in regulating the lambing number in Small-tailed Han sheep. This finding has implications for sheep breeding because it provides a valuable candidate molecular marker for marker-assisted selection in Small-tailed Han sheep. Incorporating this marker into breeding programs can help to achieve more precise genetic selection for higher lambing numbers, thereby enhancing genetic improvement and boosting the productivity of Small-tailed Han sheep populations.

## Figures and Tables

**Table 1:** Sequence information of the PCR amplification primers.

Primer Name	Primer Sequence (5′-3′)	Length (bp)
*GRHL2-SNP* (rs402967440)	F-GATGTTGCGACTGTTTGGG	19
	R-CAGAACTCCTGGGCTTTTGT	20
*GRHL2-SNP* (rs411785026)	F-CTTGGACAAAGGAAGCGGC	19
	R-GGACGACAACATCATTGAGCACT	23
*GRHL2-SNP* (rs424408342)	F-CGGAGAAAAGGTGGACAATAAAT	23
	R-TGAAAGAATGGCTGAGGGGAC	21
*GRHL2-SNP* (rs411712725)	F-AATCGTCCCCTACTCTTCCTCA	22
	R-TTGGCACCCCGCATACTTTA	20
*RORA-SNP* (rs425540123)	F-GCATCACTCATAACCTTGACCTT	23
	R-GGCATCATTTTGACATTCTCCT	22

**Table 2:** Primer sequence information of the iPLEX SNP genotyping.

Gene	Primer Name	Primer Sequence (5′-3′)	Length (bp)
*GRHL2* rs402967440 (g.75350274G > A)	Forward	ACGTTGGATGTGCTGGATGGGTGTGAGAAG	30
	Reverse	ACGTTGGATGACAAGCGTTCTCTCTCCATC	30
	Extension	CTGGCACTTCTGAAACCAGT	20
*GRHL2* rs411785026 (g.75351375T > C)	Forward	ACGTTGGATGAATGGACTGAAAGGGAGAGC	30
	Reverse	ACGTTGGATGTATTGTCCACCTTTTCTCCG	30
	Extension	CCAAAGAGTCCTGGTGGGCT	20
*GRHL2* rs424408342 (g.75351555G > A)	Forward	ACGTTGGATGGTCGTGTAACAGCAGTCGTC	30
	Reverse	ACGTTGGATGGAAATCTGAGCCCCATTCTG	30
	Extension	GGTGAGGTGGGCCTGCG	17
*GRHL2* rs411712725 (g.75371268G > A)	Forward	ACGTTGGATGCCTACTCTTCCTCATGACAG	30
	Reverse	ACGTTGGATGAAAGAAGAAGGGATGAAGCG	30
	Extension	GGGCGGGCCCCAGGGCGAGCG	21
*RORA* rs425540123 (g.46741101A > T)	Forward	ACGTTGGATGTCGAGCTACCCTTTGATCAG	30
	Reverse	ACGTTGGATGTCTGCAATTCTGGCAGCTAC	30
	Extension	AGAAAAGGCAAAGAAGGAT	19

**Table 3:** Population genetic analysis of candidate loci of *GRHL2* and *RORA* in Small-tailed Han sheep.

Gene	Loci	Genotype Frequency (Genotype Count)			Allele Frequency		*PIC*	*HE*	*NE*	Chi-Square Test (*p*-Value)
*GRHL2*	rs402967440	GG	GA	AA	G	A				
		0.96 (367)	0.04 (14)	0.01 (3)	0.97	0.03	0.05	0.05	1.05	0.00
	rs411785026	TT	CT	CC	T	C				
		0.31 (119)	0.45 (172)	0.24 (93)	0.53	0.47	0.37	0.50	1.99	0.05
	rs424408342	GG	GA	AA	G	A				
		0.93 (357)	0.03 (13)	0.04 (14)	0.95	0.05	0.10	0.10	1.11	0.00
	rs411712725	GG	GA	AA	G	A				
		0.86 (328)	0.14 (52)	0.01 (2)	0.93	0.07	0.13	0.14	1.16	0.97
*RORA*	rs425540123	AA	AT	TT	A	T				
		0.90 (345)	0.10 (38)	0.00 (1)	0.95	0.05	0.09	0.10	1.11	0.97

*NE*: number of effective alleles; *HE*: heterozygosity; *PIC*: polymorphism information content; *PIC* > 0.5 is high polymorphism, 0.25 ≤ *PIC* ≤ 0.5 is moderate polymorphism, and *PIC* < 0.25 is low polymorphism; *p* > 0.05 is in Hardy–Weinberg equilibrium; *p* < 0.05 is not in Hardy–Weinberg equilibrium.

**Table 5:** Information about the ten proteins that interact most strongly with *GRHL2*.

Description		Score
VWC2L	Von Willebrand factor C domain containing 2 like	0.654
SEC11A	Signal peptidase complex catalytic subunit SEC11	0.609
TEX2	Testis expressed 2	0.573
OVOL2	Ovo like zinc finger 2	0.571
ESRP2	Epithelial splicing regulatory protein 2	0.557
NOL10	Nucleolar protein 10	0.551
ESRP1	Epithelial splicing regulatory protein 1	0.541
TMEM182	Transmembrane protein 182	0.515
TNRC18	Trinucleotide repeat containing 18	0.513
FOXA1	Forkhead box A1	0.507

## Data Availability

Data are available on request.

## Supplementary Materials

The following supporting information can be downloaded at https://www.mdpi.com/article/10.3390/ani15101432/s1, Supplementary Table S1. Record of lambing numbers in three litters of Small-tailed Han sheep.
