# Supplementary material for: Association Analysis of SNPs in GRHL2 and RORA Genes with Lambing Number in Small-Tailed Han Sheep
Source: Animals (Basel). 2025 May 15;15(10):1432. doi: 10.3390/ani15101432 (PMC12108371; doi:10.3390/ani15101432)
Supplement: Supplementary file 1 [file animals-15-01432-s001.zip › animals-3611072-supplementary.pdf]

Supplementary Table1 Record of lambing numbers in three litters of Small-tailed Han sheep

number	1st parity lambing numbery	2nd parity lambing number	3rd parity lambing number	number	1st parity lambing numbery	2nd parity lambing number	3rd parity lambing number
1	2	2	3	101	1	0	3
2	3	0	0	102	2	1	0
3	3	3	0	103	1	0	0
4	4	2	3	104	2	0	0
5	2	1	0	105	2	0	0
6	1	3	0	106	2	0	0
7	1	2	0	107	2	4	0
8	2	0	0	108	2	2	0
9	3	3	3	109	2	0	0
10	3	3	0	110	2	0	0
11	2	5	5	111	2	0	0
12	2	3	4	112	2	0	0
13	2	2	2	113	2	0	0
14	2	3	3	114	2	0	0
15	3	0	0	115	2	2	2
16	3	4	0	116	2	0	0
17	1	2	0	117	2	0	0
18	2	2	2	118	2	0	0
19	2	3	3	119	4	3	0
20	1	2	2	120	3	0	0
21	1	0	0	121	4	0	0
22	1	1	0	122	2	0	0
23	2	3	5	123	2	0	0
24	2	2	3	124	3	3	0
25	2	3	2	125	3	2	2
26	2	0	0	126	2	2	0
27	2	3	0	127	3	0	0
28	2	2	0	128	3	0	0
29	2	3	2	129	2	0	0
30	2	3	3	130	3	3	0
31	3	0	0	131	2	0	0
32	3	0	0	132	3	0	0
33	2	0	0	133	2	0	0
34	2	2	3	134	3	0	0
35	1	0	0	135	3	0	0
36	3	0	0	136	3	0	0
37	3	3	0	137	3	0	0
38	0	1	0	138	3	0	0

39	0	2	0	139	2	0	0
40	0	2	0	140	2	0	0
41	2	0	0	141	3	2	0
42	2	0	0	142	2	3	3
43	1	0	0	143	2	2	0
44	0	2	0	144	1	2	0
45	1	0	0	145	1	0	0
46	0	2	0	146	3	2	0
47	0	2	0	147	3	0	0
48	1	0	0	148	2	1	0
49	0	1	0	149	2	0	0
50	0	1	0	150	4	2	0
51	2	0	0	151	3	2	0
52	2	0	0	152	2	0	0
53	0	1	0	153	2	0	0
54	2	0	0	154	2	0	0
55	3	0	0	155	2	0	0
56	0	2	0	156	2	0	0
57	0	1	0	157	2	2	0
58	0	1	0	158	2	2	0
59	0	1	0	159	3	0	0
60	0	2	0	160	1	0	0
61	0	2	0	161	3	0	0
62	0	1	0	162	3	3	0
63	0	1	0	163	2	0	0
64	0	3	0	164	2	0	0
65	0	1	0	165	1	0	0
66	1	0	0	166	2	3	0
67	0	1	0	167	3	4	4
68	2	0	0	168	3	3	4
69	2	0	0	169	2	3	0
70	0	2	0	170	2	0	0
71	1	0	0	171	2	2	2
72	0	2	0	172	3	3	0
73	0	2	0	173	3	3	0
74	0	2	0	174	2	3	0
75	2	0	0	175	3	3	0
76	2	0	0	176	3	3	0
77	0	3	0	177	2	2	0
78	0	2	0	178	2	2	0
79	1	0	0	179	3	3	0
80	0	2	0	180	3	0	0
81	0	2	0	181	3	3	0

82	2	0	0	182	2	0	0
83	0	2	0	183	2	3	0
84	0	2	0	184	3	3	0
85	0	2	0	185	3	0	0
86	2	0	0	186	3	0	0
87	2	0	0	187	2	3	3
88	2	0	0	188	2	3	2
89	1	0	0	189	2	3	2
90	0	2	0	190	1	2	0
91	2	0	0	191	3	3	0
92	2	0	0	192	2	3	0
93	0	2	0	193	2	2	0
94	0	2	0	194	1	2	0
95	0	2	0	195	3	4	5
96	0	3	0	196	3	3	3
97	3	3	4	197	3	4	3
98	1	0	0	198	2	3	0
99	2	0	3	199	3	1	3
100	1	0	0	200	3	3	0
number	1st parity lambing numbery	2nd parity lambing number	3rd parity lambing number	number	1st parity lambing numbery	2nd parity lambing number	3rd parity lambing number
201	3	3	4	301	3	3	0
202	2	2	0	302	2	0	0
203	2	3	0	303	3	0	0
204	2	3	4	304	2	2	4
205	1	2	1	305	3	2	0
206	2	2	3	306	2	2	0
207	4	4	5	307	2	3	3
208	3	3	0	308	3	2	0
209	3	4	3	309	2	4	2
210	3	0	0	310	2	2	2
211	2	3	0	311	4	4	4
212	2	3	2	312	4	4	4
213	3	2	3	313	4	4	4
214	3	3	4	314	4	4	4
215	2	1	2	315	4	4	4
216	0	2	3	316	4	4	4
217	4	3	0	317	4	4	0
218	2	1	5	318	4	4	4
219	2	2	0	319	2	3	3
220	2	3	0	320	3	4	0

221	2	3	0	321	3	3	0
222	2	1	2	322	1	4	3
223	2	2	3	323	1	2	1
224	2	0	0	324	1	1	0
225	2	2	0	325	1	1	2
226	2	3	2	326	2	2	2
227	2	2	0	327	2	2	3
228	1	0	0	328	2	2	2
229	2	2	0	329	2	2	0
230	2	3	0	330	2	3	0
231	1	0	0	331	4	2	0
232	2	2	0	332	1	1	2
233	3	2	0	333	3	2	0
234	1	2	3	334	2	3	0
235	3	3	3	335	2	3	0
236	2	0	0	336	2	1	0
237	1	0	0	337	2	2	0
238	2	3	0	338	2	2	0
239	3	2	0	339	4	4	0
240	1	0	0	340	2	0	0
241	2	2	2	341	1	0	0
242	2	3	0	342	1	1	1
243	3	2	0	343	2	2	2
244	1	2	2	344	3	3	0
245	3	2	0	345	1	3	2
246	2	3	0	346	1	3	2
247	1	0	0	347	1	2	0
248	2	0	0	348	1	1	0
249	1	3	0	349	1	0	0
250	2	3	0	350	3	1	0
251	1	3	0	351	1	2	0
252	2	0	0	352	2	1	0
253	2	0	0	353	2	3	0
254	3	0	0	354	1	2	0
255	2	0	0	355	2	4	0
256	2	0	0	356	3	2	0
257	2	3	6	357	2	1	0
258	3	4	3	358	1	0	0
259	3	4	0	359	2	2	3
260	2	5	3	360	1	1	0
261	2	1	0	361	2	3	3
262	4	3	3	362	1	1	0
263	3	3	0	363	2	2	0

264	3	4	5	364	1	2	0
265	3	4	3	365	4	3	3
266	2	2	0	366	3	2	0
267	2	2	0	367	3	3	0
268	3	2	0	368	2	2	3
269	2	3	0	369	4	2	2
270	2	0	0	370	2	4	0
271	2	3	3	371	2	3	1
272	2	1	1	372	1	2	0
273	2	2	3	373	2	2	0
274	2	2	0	374	2	2	0
275	2	0	0	375	2	2	2
276	2	2	0	376	1	1	3
277	1	0	0	377	2	3	0
278	1	2	0	378	1	1	0
279	2	4	0	379	1	3	0
280	2	2	2	380	1	2	0
281	5	3	0	381	1	3	3
282	4	3	4	382	1	3	0
283	2	4	0	383	2	2	0
284	3	2	0	384	2	2	0
285	3	4	0				
286	2	1	1				
287	3	4	0				
288	2	0	0				
289	3	4	0				
290	3	0	0				
291	3	2	0				
292	4	0	0				
293	4	5	5				
294	3	4	4				
295	3	0	0				
296	3	0	0				
297	2	2	0				
298	1	0	0				
299	2	0	0				
300	2	2	0				
